# Neuroplasticity in response to cognitive behavior therapy for social anxiety disorder

**DOI:** 10.1038/tp.2015.218

**Published:** 2016-02-02

**Authors:** K N T Månsson, A Salami, A Frick, P Carlbring, G Andersson, T Furmark, C-J Boraxbekk

**Affiliations:** 1 Division of Psychology, Department of Behavioural Sciences and Learning, Linköping University, Linköping, Sweden; 2 Department of Adult Psychiatry, PRIMA Barn och Vuxenpsykiatri, Stockholm, Sweden; 3 Department of Neurobiology, Care Sciences and Society, Aging Research Center, Karolinska Institutet, Stockholm, Sweden; 4 Umeå Center for Functional Brain Imaging, Umeå University, Umeå, Sweden; 5 Department of Psychology, Uppsala University, Uppsala, Sweden; 6 Department of Psychology, Stockholm University, Stockholm, Sweden; 7 Department of Clinical Neuroscience, Psychiatry Section, Karolinska Institutet, Stockholm, Sweden; 8 CEDAR, Center for Demographic and Aging Research, Umeå University, Umeå, Sweden

## Abstract

Patients with anxiety disorders exhibit excessive neural reactivity in the amygdala, which can be normalized by effective treatment like cognitive behavior therapy (CBT). Mechanisms underlying the brain's adaptation to anxiolytic treatments are likely related both to structural plasticity and functional response alterations, but multimodal neuroimaging studies addressing structure–function interactions are currently missing. Here, we examined treatment-related changes in brain structure (gray matter (GM) volume) and function (blood–oxygen level dependent, BOLD response to self-referential criticism) in 26 participants with social anxiety disorder randomly assigned either to CBT or an attention bias modification control treatment. Also, 26 matched healthy controls were included. Significant time × treatment interactions were found in the amygdala with decreases both in GM volume (family-wise error (FWE) corrected *P*^FWE^=0.02) and BOLD responsivity (*P*^FWE^=0.01) after successful CBT. Before treatment, amygdala GM volume correlated positively with anticipatory speech anxiety (*P*^FWE^=0.04), and CBT-induced reduction of amygdala GM volume (pre–post) correlated positively with reduced anticipatory anxiety after treatment (*P*^FWE^⩽0.05). In addition, we observed greater amygdala neural responsivity to self-referential criticism in socially anxious participants, as compared with controls (*P*^FWE^=0.029), before but not after CBT. Further analysis indicated that diminished amygdala GM volume mediated the relationship between decreased neural responsivity and reduced social anxiety after treatment (*P*=0.007). Thus, our results suggest that improvement-related structural plasticity impacts neural responsiveness within the amygdala, which could be essential for achieving anxiety reduction with CBT.

## Introduction

The brain is remarkably adaptive. Neuroimaging studies in animals and humans have shown multiple facets of plasticity,^1, 2^ manifested as structural changes induced by environment,^3^ learning,^4^ behavior^1, 5^ and emotions.^6^ Similar to structural plasticity, emotions alter neural responsivity.^7, 8^ Although the notion that the human brain is adaptive has extensive support from unimodal brain imaging studies, integrative models of the brain's functional changes in relation to structural plasticity are largely lacking, and multimodal studies are needed to improve our understanding of the adaptive brain.^9, 10^ For instance, using a longitudinal multimodal-imaging approach, Nyberg and colleagues^11, 12^ demonstrated that age-related reductions in prefrontal functional response to a cognitive task are driven by local gray matter (GM) atrophy. The observation that neural response alterations over time are accounted for by structural changes may have implications for research in psychiatric disorders. For example, a recent large study found that both GM volume and neural responsivity in the amygdala were associated with symptoms of separation anxiety,^13^ suggesting dependence between anxiety-related brain structure and function.

The amygdala, anterior cingulate cortex (ACC), insula and hippocampus, have central roles in the acquisition and expression of fear.^14^ Exaggerated neural response in these regions has commonly been reported in anxiety disorders.^15, 16, 17^ In comparison to functional activation studies, volumetric differences between anxiety patients and healthy controls have been investigated less frequently,^18, 19, 20, 21, 22, 23, 24^ and inconsistent findings are present, for example, in volumetric studies on social anxiety.^25^ Examinations of both structure and function concomitantly in anxious patients are largely lacking,^26^ and it has not been studied whether anxiety-related structure and neural activity are simultaneously changed by effective treatments. Treatments targeting anxiety, like cognitive behavior therapy (CBT)^27, 28, 29, 30, 31^ and psychotropic medication (for example, with selective serotonin reuptake inhibitors),^28, 32^ have been shown to decrease neural responsivity in the amygdala. In line with this, Mahan and Ressler^33^ suggested that synaptic plasticity in the amygdala may be an important target for treatment of posttraumatic stress disorder. While there is limited literature on structural changes with anxiety-reducing pharmacologic agents^34, 35^ or psychotherapy,^36^ the results are mixed and the conclusions are restricted by not including a treatment control group.

The present randomized controlled trial (RCT) examined CBT-related changes in the brain using a multimodal neuroimaging approach. Hence, we evaluated the relationship between structural neuroplasticity (GM volume) and functional changes (blood–oxygen level dependent, BOLD responsivity to self-referential criticism)^37^ in participants with social anxiety disorder (SAD) who were treated with CBT or an attention-training control treatment.^27^ Brain parameters were also related to a matched healthy control group to evaluate pre-treatment differences and normalization effects of treatment. We expected concomitant treatment-related changes in GM volume and functional response in the brain's fear circuitry (that is, the amygdala, ACC, insula and hippocampus).^17^ Mediation analysis was conducted to determine the path for reduced social anxiety after treatment, that is, whether structural neuroplasticity or altered neural response mediated the relationship between the other and symptom improvement with CBT.

## Materials and methods

### Participants

Fifty-two right-handed participants were included (see Table 1), 26 with a primary diagnosis of SAD according to the structured clinical interview for DSM-IV axis I (SCID-1)^38^ and 26 healthy controls matched on age, sex and educational level, and free from psychiatric disorders as assessed by the Mini-International Neuropsychiatric Interview (MINI).^39^ Prior to the diagnostic telephone interview, participants answered Internet-administered self-report questionnaires regarding social anxiety (for example, Liebowitz Social Anxiety Scale – Self-report version (LSAS-SR)^40^ see online Supplementary Material), depression (for example, Montgomery–Åsberg Depression Rating Scale—Self-report version (MADRS-S))^41^ and magnetic resonance safety. Eight SAD participants were on prescription medication throughout the study, but the selective serotonin reuptake inhibitors dose had been stable for at least 3 months prior to treatment initiation. As shown in Table 1, SAD participants had higher scores on social anxiety and depressive symptoms than the healthy controls.

The study was registered at ClinicalTrials.gov (id: NCT01312571), and approval was obtained from the regional ethics committee. All participants gave written informed consent prior to participation.

### Procedure and design

The present paper is part of a RCT previously described in detail.^27, 42^ In short, participants with SAD were treated with Internet-delivered CBT or Internet-delivered Attention Bias Modification (ABM), and an independent researcher executed the randomization. Magnetic resonance imaging (MRI) assessments were performed before as well as 9 weeks after treatment (pre–post). The matched healthy controls underwent only one MRI assessment. The SAD participants were not offered economic compensation, but the healthy controls received about 125 USD and a cinema ticket.

### Clinical assessment

An independent clinical psychologist, blind to the experimental conditions, determined clinical response rates using the Clinical Global Impression-Improvement scale (CGI-I; scores 1 or 2, that is, much or very much improved defining treatment responders).^43^ Moreover, social anxiety self-report questionnaires were administrated at pre- and post-treatment (for example, LSAS-SR).^40^ We were also interested in state-related social anxiety,^44^ so after the MRI assessment the participants performed a 2-min public speaking task. Both after the initial anatomical image acquisition, and prior to the speech, the participants rated subjective units of discomfort. Fear and distress were rated separately on a scale from 0 to 100 (min–max), and anxiety was calculated as the mean of these measures.

### Treatment

The CBT in the present study was delivered over a period of 9 weeks, and the therapist provided written feedback once a week. Several independent RCTs on guided Internet-delivered CBT for SAD show robust effects,^45, 46^ and comparable improvement to conventional CBT delivered face-to-face.^47^ We have previously demonstrated that the present treatment, compared with ABM, reduced anxiety-related amygdala responsivity to emotional faces.^27^

ABM is a computer-assisted intervention aimed at improving a threat-detecting cognitive bias that characterizes SAD.^48^ In concordance with previous studies, the ABM was delivered twice a week over 4 weeks. ABM has been described in detail elsewhere.^27, 42, 49^ While ABM has shown promise,^50, 51^ it has been found to be less effective when delivered through the Internet^49^ and we used ABM as a control in the present trial. See online Supplementary Material for further details.

### BOLD-fMRI experimental task

BOLD response to self-referential criticism was recorded using functional magnetic resonance imaging (fMRI) and an information-processing paradigm developed by Blair *et al.*^37^ Essentially, the participants were instructed to read sentences and press a button using their right hand as a confirmation. The sentences contained criticism targeting the participant or others. Functional responses to self and other referential information were recorded for a maximum of 2500 ms, and fixation crosses (‘+' 2500 ms) were randomly interspersed between the sentences. In addition, each sentence and fixation cross was separated by a cross or circle presented for 500 ms (see also Supplementary Figure S3). Stimuli were demonstrated using the E-prime 2.0 software (Psychology Software Tools, Pittsburgh, PA, USA), projected on a screen and viewed through a tilted mirror attached to the head coil. For more details, see the study Månsson *et al.*^42^

### Image acquisition and preprocessing

Neuroimaging was performed in a 3 T scanner (General Electric, Madison, WI, USA), equipped with a 32-channel head coil. Structural T1-weighted images were acquired with voxel size: 0.5 × 0.5 × 1 mm^3^ (180 slices; field of view: 250 mm). For functional images, the following parameters were used: echo time: 30 ms, repetition time: 2000 ms, flip-angle: 80°, field of view: 250 × 250 mm^2^, matrix size: 96 × 96, in-plane resolution: 2.6 × 2.6 mm. Thirty-seven slices with a thickness of 3.4 mm were acquired every 2000 ms. Ten dummy scans were run before the image acquisition started to avoid signals resulting from progressive saturation.

The Statistical Parametric Mapping Software v. 8 (SPM8; Wellcome Department of Cognitive Neurology, London, UK) and the MATLAB (Mathworks, Natick, MA, USA) were used to perform neuroimaging analyses. The T1-weighted images were preprocessed using the VBM8 toolbox (http://dbm.neuro.uni-jena.de/vbm/download). The Voxel-Based Morphometry v. 8 (VBM8) toolbox calculates modulated normalized GM volumes and allows for comparing tissue amounts while controlling for individual brain sizes (default settings were used). To identify outliers, quality control was carried out using the sample homogeneity test, and we found that covariance was within 2 s.d. Thus, no outliers were excluded. VBM8 preprocessing was performed in 3 steps; (a) longitudinal MRI data assessment on SAD participants (pre- vs post-treatment; *n*=23 × 2; that is, three participants withdrew from the post-MRI assessment), (b) case–control differences at baseline (SAD vs healthy control; *n*=26+26) and (c) case–control differences following CBT (*n*=11+26). Structural scans were segmented into gray and white matter, and the GM volumes were non-linearly normalized to the Montreal Neurological Institute (MNI) template, modulated and smoothed using an 8 mm full-width half-maximum isotropic Gaussian kernel.

Functional MRI data were first rigidly aligned to the middle image volume of each run to correct for head movements. The realigned images were then corrected for acquisition time differences between slices within each volume. A within-subject rigid registration was conducted to align functional and structural images together. For the 23 SAD participants who underwent the post-treatment MRI, we co-registered the functional scans to a longitudinal mean structural image (if post-treatment images were missing, the pre-treatment image was used). For healthy controls, the functional scans were co-registered to the structural image. Functional scans were subsequently warped to MNI152 standard space (using the transformation parameters that normalized GM images into the MNI space) and smoothed with an 8 mm full-width half-maximum isotropic Gaussian kernel. Thus, both fMRI and VBM images were in the same space and had the same voxel size (that is, 1.5 × 1.5 × 1.5 mm^3^).

In the BOLD-fMRI paradigm, subject-specific contrasts (self-referential criticism vs other referential criticism) were generated with voxel-wise general linear models. Each condition was modeled as a box-car function, convolved with the hemodynamic response function and filtered using a 128 s high-pass filter. In addition, six motion parameters derived from the realignment algorithm were included in the model to account for motion artifacts.

### Data analysis

Demographics, clinical data and mediation analyses were evaluated using the STATA Statistical Software, v. 13.1 (STATA, College Station, TX, USA) and SPSS Statistics, v. 19.0 (IBM, Armonk, NY, USA).

Regions of interest (ROI) in all neuroimaging analyses were the left and right amygdala, ACC, insula and hippocampus, that is, regions of the fear circuitry^17^ affected in SAD.^15, 16, 25, 52^ ROIs were defined using the AAL Atlas from the Wake Forest University PickAtlas,^53^ and the significance threshold level was set at *P*<0.05 with family-wise error (FWE) correction on voxel-wise comparisons within each ROI. Whole-brain analyses used *P*<0.001 as the significance threshold and 10 voxels as the extent threshold.

The clinical outcome of CBT, relative to the ABM, on the CGI-I was calculated using a *χ*^2^-test, and self-reported effects were evaluated using repeated measure MANOVA (multivariate analysis of variance on social anxiety questionnaires) or MANCOVA (multivariate analysis of covariance on state-related anxiety).

Time (pre- and post-treatment) × treatment (CBT and ABM) interactions were assessed using the flexible factorial design. Significant interactions were further qualified with pairwise comparisons, that is, paired *t*-tests within the CBT group. Each imaging modality was analyzed separately. The association between social anxiety (that is, LSAS-SR and anticipatory speech anxiety) and either GM volume or BOLD responsivity was calculated in separate regression models, both at pre-treatment and as a function of CBT. Change in GM volume (ΔGM), and BOLD response (ΔBOLD) were calculated using the SPM8 ImCalc function. To assess case–control differences in structure and function, as well as putative normalization effects of CBT, we compared GM volume and BOLD responsivity (at pre- and post-treatment) in SAD participants relative to healthy controls using independent two-sample *t*-tests within each image modality.

To explore the most probable path for reduced social anxiety after treatment, that is, structural neuroplasticity or change on neural response, we performed mediation analyses in accordance with the Shrout and Bolger model,^54^ in which sufficient statistical power may be less critical to detect mediation. The outcome measure used in these analyses was social anxiety symptom improvement, that is, changes either in LSAS-SR scores or anticipatory speech anxiety (using separate models). First, we extracted changes in GM volume and BOLD response from voxels exhibiting significant positive correlations *P*<0.05 (FWE corrected at cluster-level) between these measures (the *a*-path). Correlations were established using the Biological Parametric Mapping (BPM) toolbox.^55^ BPM is a multimodal-imaging approach that can model change in one modality (for example, BOLD-fMRI) as a function of change in another modality (for example, GM volume) using the general linear model framework. The *b*-path refers to the relationship between the mediator and the outcome (that is, social anxiety improvement). We calculated mediation paths with either change in GM, or change in BOLD response as mediators. The interaction of *a*- and *b*-path represents the indirect effect, and the predictor–outcome association is referred to as the direct effect (the *c'*-path). The interaction term was estimated using bootstrap resampling (*n*=5000) to jointly estimate the direct and indirect effects, therefore minimizing the dependence on normally distributed data.^54^

## Results

### Treatment outcome and compliance

As previously reported,^27^ the clinician administrated CGI-I assessments revealed significantly more participants responding positively to the CBT (61%, 8/13) than to the ABM control treatment (23%, 3/13; *χ*^2^=3.94, *P*=0.047), and on the self-report questionnaires, we found similar results in favor of CBT.^27^ Furthermore, when controlling for pre-treatment level, anticipatory speech anxiety decreased more with CBT than with ABM (time × treatment; Wilks's *λ*=0.678, *F*_2,21_=4.98, *P*=0.017), see Supplementary Table S1, and Supplementary Figure S4.

On average, the CBT participants completed eight (out of nine) modules of treatment. In addition to completing the module-based assignments, the participants were required to earn at least 95% correct answers on a multiple-choice quiz about CBT every week. The ABM control participants earned an average of 98.4% (16 383/16 640) correct responses on the attentional shifting task, and they completed all training sessions.

### Treatment effects on brain structure and neural responsivity

**Figure 1:**
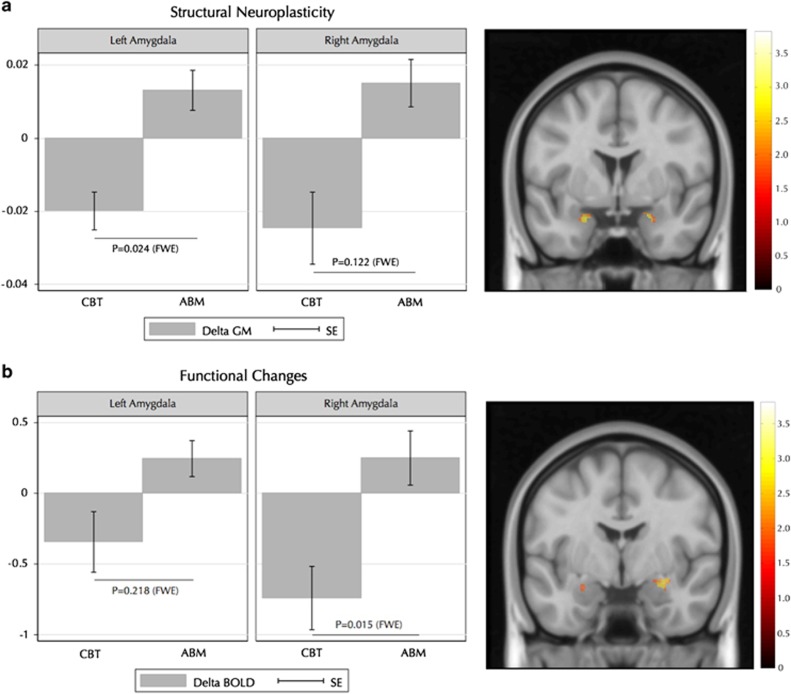
Changes in (**a**) amygdala GM volume and (**b**) amygdala BOLD response to self-referential criticism, sorted by treatment and hemisphere. The *y*-axis shows change in beta-weight values, and lower beta-weights correspond to reduced GM volume and BOLD responsivity over time (pre–post). Error bars represent s.e. ABM, attention bias modification; CBT, cognitive behavior therapy; FWE, family-wise error corrected *P*-value; GM, gray matter volume.

Time × treatment interactions indicated that left amygdala GM volume and right amygdala BOLD response decreased significantly more with CBT compared with the ABM control treatment (see Table 2 and Figure 1). Similarly, follow-up pairwise comparisons within the CBT group suggested decreased left amygdala GM volume and right amygdala BOLD responsivity after treatment (see Table 2).

Whole-brain analysis of structural change showed that the GM volumes of the dorsomedial prefrontal cortex (Brodmann area 8) and the bilateral precuneus were more greatly reduced after CBT than ABM (see Supplementary Table S2). Whole-brain BOLD-signal analyses only revealed a greater reduction in the activation of the right amygdala (*xyz*: 29,1,–16; *Z*=3.28, *P*<0.001, *k*=15 voxels) in CBT compared with ABM participants.

### Relationship between social anxiety symptomatology, brain structure and neural responsivity

**Figure 2:**
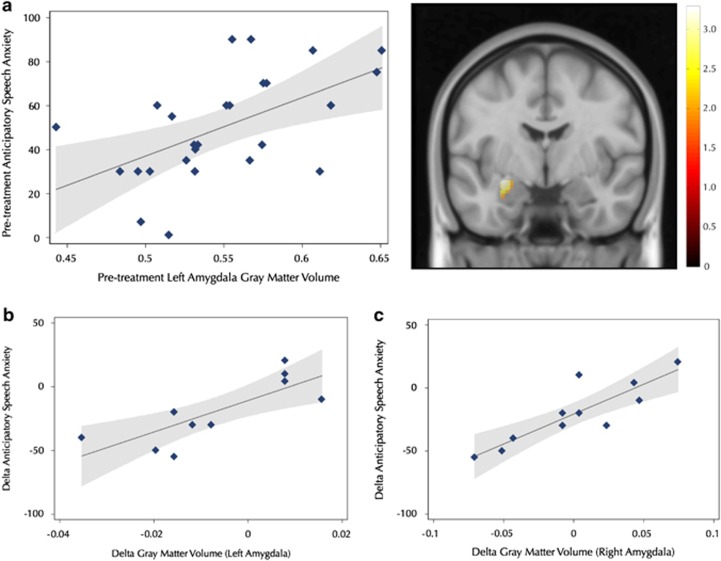
Brain-behavior correlations showing that (**a**) pre-treatment gray matter volume in the left amygdala was associated with enhanced levels of anticipatory speech anxiety, and (**b** and **c**) reduced amygdala gray matter volume (left and right respectively) was positively associated with improved symptoms of anticipatory speech anxiety after cognitive behavior therapy. Gray shading corresponds to 95% confidence intervals.

Pre-treatment GM volume in the left amygdala was positively correlated with the level of anticipatory speech anxiety in SAD participants (*xyz*: –24,–4,–12; *Z*=2.96, *P*^FWE^=0.04; *k*=273 voxels, see Figure 2). As reported in Supplementary Table S3, whole-brain analysis additionally showed that GM volume of the left and right precuneus was positively correlated with anticipatory speech anxiety.

CBT-induced reductions of the GM volumes of the bilateral amygdala and the insula were positively associated with decreased levels of anticipatory speech anxiety (left amygdala, *xyz*: –16,–3,–18; *Z*=2.83, *P*^FWE^=0.05, *k*=73 voxels; right amygdala, *xyz*: 22,6,–18; *Z*=3.42, *P*^FWE^=0.01, *k*=131 voxels; see Figure 2; left insula, *xyz*: –40,14,1; *Z*=4.02, *P*^FWE^=0.02, *k*=468 voxels; right insula, *xyz*: 28,12,–18; *Z*=3.69, *P*^FWE^=0.04, *k*=128 voxels). Whole-brain analysis revealed anxiety-related reductions of the GM volume in the left fusiform gyrus (Supplementary Table S4).

Contrary to our expectations, GM volume was not associated with general social anxiety symptomatology as measured with LSAS-SR; nor was BOLD responsivity significantly correlated with pre-treatment severity or pre–post improvement of symptoms (anticipatory speech anxiety or LSAS-SR).

### Comparisons of brain structure and neural response between SAD participants and healthy controls

Before treatment there was no significant difference between SAD participants and healthy controls in GM volume within the fear neurocircuitry (*Z*<2.99, *P*^FWE^>0.374). Amygdala BOLD response to self-referential criticism was elevated in participants with SAD compared with the healthy controls (right amygdala, *xyz*: 27,–8,–12; *Z*=3.04, *P*^FWE^=0.029, *k*=150 voxels; with a trend in the left amygdala (*xyz*: –26,–2,–11; *Z*=2.54, *P*^FWE^=0.088, *k*=90 voxels). The post-CBT amygdala response did not differ significantly from that of healthy controls (*Z*<.50, *P*^FWE^>0.743), indicating normalization through CBT. Result from the whole-brain analysis is reported in Supplementary Table S5.

### Mediation analysis

Mediation analysis was conducted to determine the most probable brain path for improvement in anticipatory speech anxiety. Within the CBT group, the *a*-path was significant in both amygdalae, indicating that structural plasticity was interrelated with diminished amygdala neural responsivity (right amygdala, *xyz*: 29,1,–22; *Z*=3.14, *P*^FWE^=0.001 at cluster-level, *k*=71 voxels; left amygdala, *xyz*: –21,2,–20; *Z*=2.45, *P*^FWE^=0.029 at cluster-level; *k*=40 voxels).

**Figure 3:**
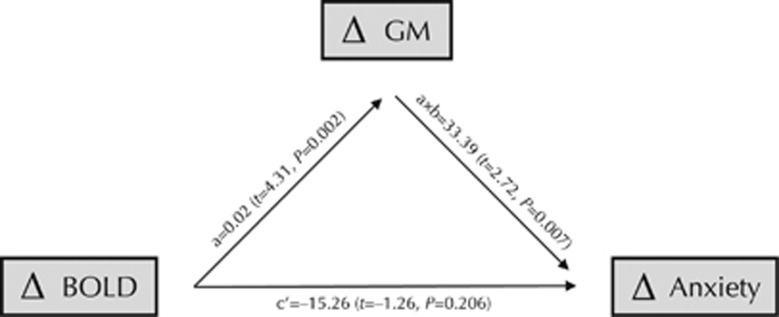
The mediation paths supported reduced gray matter volume as the mediator of the relation between diminished amygdala BOLD responsivity (predictor) and improved anticipatory speech anxiety after cognitive behavior therapy. GM, gray matter.

In the right amygdala, reduced GM volume mediated the relationship between reduced BOLD response and symptom improvement (*a* × *b*-path, indirect effect: *β*=33.39, 95% CI=9.32 to 57.45, *P*=0.007). The direct effect was not significant (*c'*-path: *β*=–15.26, 95% CI=–38.93 to 8.40, *P*=0.206; see also Figure 3). Furthermore, without controlling for the indirect effect, the *c*-path suggested an opposite relationship (*β*=18.12, *P*=0.104) between the predictor and the outcome, that is, an inverse association. The other probable path, that is, BOLD response as mediator, was not significant (*β*=–479.90, 95% CI=–1469.93 to 510.14, *P*=0.342).

### Supplementary analyses

As detailed in the online Supplementary Materials, we found no pre-treatment differences in clinical and demographic (for example, age and sex) variables, nor did the amygdala characteristics (that is, GM volume or BOLD responsivity) differ between participants allocated to CBT or ABM. Furthermore, we found no clinical, or demographic differences between included participants and the three participants who withdrew from the post-treatment MRI assessment. Finally, we performed an alternative mediation analysis using the CGI-I responder status as the outcome measure, noting a trend-level indirect effect (*a* × *b*-path).

## Discussion

Using a multimodal neuroimaging RCT design, we demonstrate interrelated structural plasticity and altered neural responsivity, within the amygdala, after CBT for social anxiety. Both GM volume and neural responsivity in the bilateral amygdala diminished after effective treatment. Left amygdala GM volume was positively associated with symptom severity before treatment, and amygdala volume decreased significantly with CBT, correlating positively with symptom improvement in both hemispheres. Concomitantly, amygdala hyperresponsivity to self-referential criticism was normalized with CBT, and the mediation paths suggested that reduced amygdala volume mediated the relationship between decreased right amygdala neural response and decreased social anxiety after treatment.

Thus, we demonstrate that the relationship between CBT-induced attenuation of amygdala hyperresponsivity and social anxiety symptoms is mediated by decreased local GM volume. Similar to our finding on the structure–function relationship in the amygdala, a previous longitudinal study also showed dependence between GM atrophy and age-related cognitive neural responsivity in the prefrontal cortex,^11^ suggesting that the adaptive brain may be best understood in a multimodal context. Hence, we argue that analyses of structural neuroplasticity and concomitant functional changes provide better understanding of how the brain adapts to anxiolytic treatments, which could not be fully explained by each modality separately. Furthermore, our results reinforce the notion that structural neuroplasticity in the amygdala is an important target for psychosocial treatments of anxiety, as previously suggested for pharmacological treatments of posttraumatic stress disorder.^33^

In our whole-brain analyses we found volumetric reduction, but not decreased neural response, following CBT in regions that have been linked to self-referential processing and commonly also in studies of the default mode network (that is, the dorsomedial prefrontal cortex and precuneus),^56^ see Supplementary Tables S2-S4. Resting-state fMRI and neural response in the default mode network have also been suggested to be biomarkers for SAD.^57^ Further, as suggested in a previous BOLD-fMRI study on self-referential criticism,^37^ SAD participants were hyperresponsive in the amygdala. The present results further indicate that the excessive amygdala responsivity is normalized with successful CBT.^27, 28, 29, 58^ This is consistent with studies demonstrating reduced amygdala responsivity after anxiolytic treatments,^29, 30, 31, 32, 44, 59, 60, 61^ but because individuals with SAD show residual symptoms of social anxiety after effective treatment, normalized neural response in isolation may not solely explain reduced symptom severity. This further underscores the importance of taking multimodal measures of brain function and structure into account.^10^

The interrelationship between neuronal response changes and the underlying anatomical plasticity is not clearly established in the animal literature.^62, 63^ However, a large body of research exists on fear and anxiety in the brain,^8, 64, 65^ suggesting that both the number of recruited amygdala neurons^14^ and the strength of the neuronal response correlate with anxious behavior in rats.^66^ Trabalza *et al.*^2^ showed structural rearrangements in the mice amygdala, the density of spines and number of nodes increasing after fear conditioning. Chronic stress also induces the formation of new synapses in the amygdala.^67^ Similarly, the total number and the size of synapses are reversed during fear extinction,^68, 69^ that is, the laboratory analog to exposure interventions in CBT.^70^ Thus, we speculate that the attenuated anxiety-related amygdala volume could be due to synaptic reorganization, such as changes in spine shape or density, or a reduced number of synapses. However, in the present study the morphological neuroplasticity was related only to decreased state-dependent (anticipatory speech) anxiety, and not to more enduring symptoms of social anxiety as measured with the LSAS-SR.

Although the RCT design and multimodal-imaging approach are noteworthy strengths of the present study, there are also a number of limitations. As discussed elsewhere,^27, 42^ the number of participants in the present study is limited, yet, sufficient to detect a differential treatment effect between CBT and ABM. The mediation analysis is also limited by not including a second repeated MRI assessment, that is, predictor and mediator were assessed at the same time-point. In addition, structural neuroplasticity and functional changes were closely related, but they may still be independently controlled by other processes such as metabolism,^71, 72^ or the corticotropin-releasing factor system.^3^ Reduced amygdala neural response after CBT did not correlate with improved social anxiety. It is, however, likely that the task-specific neural activations to self-referential criticism target cognitions not covered in our measures of state-dependent anxiety or LSAS-SR symptoms. Finally, eight SAD participants were on concurrent psychotropic medication, so we cannot entirely rule out drug × treatment interactions. However, these participants were evenly distributed in the trial arms.

In conclusion, we demonstrate compelling evidence that CBT for a common anxiety disorder simultaneously changes the physical structure and neurofunctional response of the amygdala. While our results support that amygdala neuroplasticity is directly related to improved social anxiety symptoms with CBT, these results should be replicated and further tested in other anxiety disorders and with other anxiolytic treatments.

## Figures and Tables

**Table 1:** Demographic and clinical characteristics of SAD participants and healthy controls

	*SAD participants (*n*=26)*	*Healthy controls (*n*=26)*	*Between-group statistics (*n*=52)*
Age, years, mean (s.d.)	32.27 (9.7)	32.23 (10.5)	*t*(51)=0.01, *P*=0.989
Range, years	19–57	18–57	—
Gender, female (%)	22 (85)	18 (69)	*χ*^2^=1.73, *P*=0.188
*Highest educational level*, n (%)			*χ*^2^=0.42, *P*=0.810
Completed university	9 (35)	11 (42)	
Current university	10 (38)	8 (31)	
Lower grade^a^	7 (27)	7 (27)	
Age of SAD onset, years, mean (s.d.)	15.88 (6.0)	—	
LSAS-SR, mean (s.d.)	76.31 (18.7)	20.53 (11.4)	*t*(51)=12.97, *P*<0.001
MADRS-S, mean (s.d.)	15.73 (6.6)	6.27 (4.9)	*t*(51)=5.87, *P*<0.001
Anticipatory speech anxiety, mean (s.d.)	50.15 (24.1)	13.44 (16.0)	*t*(51)=6.48, *P*<0.001

Abbreviations: LSAS-SR, Liebowitz Social Anxiety Scale—Self-report version; MADRS-S, Montgomery Åsberg Depression Rating Scale—Self-rating version; SAD, social anxiety disorder.

a Including high school, vocational school and compulsory school.

**Table 2:** Structural and functional response alterations in participants treated with effective CBT, in comparison to the ABM control treatment (time × treatment interactions)

*Analyses and brain regions*	*MRI*	*MNI coordinates*			*Maximum*	*Voxels*	P^*FWE*^
		x	y	z	Z *value*		
*2 × 2 interactions (pre-treatment vs post-treatment × CBT vs ABM)*							
L Amygdala	GM	–20	–1	–21	3.30	69	0.024
R Amygdala	GM	22	2	–21	2.70	27	0.122
L Amygdala	BOLD	–26	–7	–17	2.05	26	0.218
R Amygdala	BOLD	28	0	–16	3.28	140	0.015

*Main effects of CBT (pre-treatment>post-treatment)*							
L Amygdala	GM	–20	–1	–21	3.12	61	0.060
R Amygdala	GM	22	2	–21	2.15	17	0.395
L Amygdala	BOLD	—	—	—	<0.0		
R Amygdala	BOLD	28	2	–16	2.89	78	0.061

Abbreviations: ABM, attention bias modification; CBT, cognitive behavior therapy; FWE, family wise-error-corrected *P*-value; GM, gray matter volume; MNI, Montreal Neurological Institute template
