# Supplementary material for: Neuroplasticity in response to cognitive behavior therapy for social anxiety disorder
Source: Transl Psychiatry. 2016 Feb 2;6(2):e727–. doi: 10.1038/tp.2015.218 (PMC4872422; doi:10.1038/tp.2015.218)
Supplement: Supplementary Information [file tp2015218x1.docx]

Online Supplementary Material

**Neuroplasticity in Response to Cognitive Behavior Therapy for Social Anxiety Disorder**

Kristoffer N.T. Månsson^a,b^*, MSc

Alireza Salami^c,d^, PhD

Andreas Frick^e^, MSc

Per Carlbring^f^, PhD

Gerhard Andersson^a,g^, PhD

Tomas Furmark^e^, PhD

Carl-Johan Boraxbekk^d,h^, DrMedSc

^a^ Department of Behavioural Sciences and Learning, Division of Psychology, Linköping University, Linköping, Sweden

^b^ PRIMA Psychiatry, Stockholm, Sweden

^c^ Aging Research Center, Karolinska Institutet, Stockholm, Sweden

^d^ Umeå Center for Functional Brain Imaging, Umeå University, Umeå, Sweden

^e^ Department of Psychology, Uppsala University, Uppsala, Sweden

^f^ Department of Psychology, Stockholm University, Stockholm, Sweden

^g^ Department of Clinical Neuroscience, Psychiatry Section, Karolinska Institutet, Stockholm, Sweden

^h^ CEDAR, Center for Demographic and Aging Research, Umeå University, Umeå, Sweden

The authors declare no conflict of interest.

*Address for correspondence: Linköping University, IBL, Psychology, SE-581 83 Linköping, Sweden. Tel: +46 (0)70 5085082; fax: +46 (0)13 282145, E-mail address: kristoffer.nt.mansson@liu.se (K.N.T. Månsson).

Supplementary MATERIALS AND Method

Clinical outcomes

Clinician administrated telephone interviews were performed to determine responder status according to the Clinical Global Impression-Improvement (CGI-I) scale.[^1^](#_ENREF_1) Two participants did not partake in the CGI-I assessment, and they were accordingly categorized as “nonresponders”. Additionally, fulfilling the diagnostic criteria for SAD at post-treatment (yes/no), as determined with the Structured Clinical Interview for DSM-IV Disorder (SCID) interview[^2^](#_ENREF_2), was used as a more conservative measure of treatment outcome.

After the magnetic resonance imaging (MRI) scanning sessions, the participants rated their state-related anxiety (fear and distress, 0-100, min-max).[^3^](#_ENREF_3) Participants were also asked to perform a public speaking task (see Figure S1), and they rated anticipatory speech anxiety prior to the speech.

Procedure and design

The flow-chart diagram in Figure S2 shows participant information including random assignment and attrition details.

Validation of the fMRI experimental task

The functional magnetic resonance imaging (fMRI) experimental task (see Figure S3) has been described in detail elsewhere.[^4^](#_ENREF_4) The task was translated into Swedish (originally in English),[^5^](#_ENREF_5) and tested in a separate sample of adults (*n*=121). This group of participants was not included in MRI scanning. Specifically, we were interested if participants rated each valence of self-referential information (i.e., negative, neutral, and positive) differently in terms of how each sentence made them feel. The participants rated each statement on a 7-point scale, ranging from extremely unhappy to extremely happy.

Psychological treatments

*Internet-delivered cognitive behavior therapy*

In the present study we used a well-documented, evidence-based cognitive behavioral treatment program for SAD.[^6-9^](#_ENREF_6) The online intervention program is delivered over a period of 9 weeks, with weekly e-mail contact with a clinical psychologist who provides guidance and feedback. The psychologist provided a new treatment module every week, and to ensure compliance, the participants had to complete homework assignments and multiple-choice quizzes before moving on to the next treatment module. The participants had to get at least 95% of the answers correct on the quizzes. The psychologist feedback was designed to reinforce and augment the patient´s work throughout treatment. Weekly feedback was administered during individual 15-minute sessions, and the psychologist provided all feedback via text in the web-platform.

As described in detail elsewhere,[^7^](#_ENREF_7) the first module contained an introduction to CBT and SAD. Modules 2-4 described the cognitive model for SAD,[^10^](#_ENREF_10)^,^ [^11^](#_ENREF_11) and modules on cognitive restructuring were also included. Between the 5^th^ and 7^th^ treatment modules, we sought to motivate the SAD participants to perform exposure-based exercises (i.e., enter anxiety provoking social situations and remain until habituation occurs). Before treatment termination we also provided information on social skill training and relapse prevention (i.e., module 8-9). The treatment program is published in Swedish as a self-help book.[^12^](#_ENREF_12)

*Internet-delivered attention bias modification*

The Internet-delivered attention bias modification (ABM) was used as a control treatment in the present study and it is described in more detail elsewhere.[^13^](#_ENREF_13) Briefly, ABM is a computer-assisted intervention aimed at improving a threat-detecting cognitive bias that characterizes SAD.[^14^](#_ENREF_14) Two pictures of the same person making different facial expressions were displayed in a vertical alignment for 500 milliseconds on the participant’s computer screen. In total, 160 trials (pairs of faces) were displayed each training session, including 128 neutral and disgust facial expression pairs. For these trials, a probe appeared by the neutral face, and the participants were instructed to indicate whether the probe was the letter E or F by pressing (as fast as possible) the corresponding arrow on the keyboard using their right hand. The probe remained on the screen until a response was given, after which the next trial began. The ABM was delivered twice a week for 4 weeks in total.

Data analysis

Differences between ratings on the self-referential valences task were detected and analyzed using 2-sample *t*-test´s (i.e., negative vs. neutral, and negative vs. positive).

Participants assigned to CBT and participants assigned to ABM were compared in terms of clinical and demographic variables, gray matter (GM) volume, and blood-oxygen level-dependent contrast imaging (BOLD-fMRI). The analyses were performed using 2-sample *t*-test´s, or χ^2^ test´s on nominal scales. Withdrawal analyses, including pre-treatment clinical and demographic variables (determined using 2-sample *t*-test´s, or Fisher´s Exact test), were done on the 3 participants who did not undergo post-treatment MRI assessments. The number of participants fulfilling SAD diagnostic criterion after CBT and ABM (between-groups) was determined using a χ^2^ test.

In addition to the region of interest (ROI) approach, voxel-wise whole-brain calculations (*P*<0.001, cluster extent exceeding 10 voxels) were performed on all the neuroimaging data (e.g., treatment effects, and case-control comparisons).

Furthermore, using the post-treatment CGI-I responder status as the outcome variable, we did an additional mediation path analysis. We also performed similar mediation path analyses using the participant data from the ABM control treatment.

Supplementary Results

Validation of the fMRI experimental task

Sentences containing self-referential criticism (M=2.45, SD=1.02) were perceived more negatively compared to both neutral (M=4.60, SD=0.48) and positive sentences (M=5.47, SD=0.66; 2-sample *t*-test´s: *t*(120)>21.80, *P*<0.001).

Pre-treatment differences and withdrawal analyses

We found no pre-treatment clinical or demographic (i.e., age, educational level and sex) dissimilarities between the SAD participants in the two trial arms (*P*>0.84). Correspondingly, pre-treatment amygdala GM volume and BOLD responses did not differ between CBT and ABM participants at a corrected *P*-level (*P*^uncorr^≥0.04).

We found no clinical, or demographic differences between participants included in the study, and those (*n*=3) who withdrew from the post-treatment MRI assessment (*P*>0.26).

Treatment effects on social anxiety

As reported elsewhere,[^15^](#_ENREF_15) we found a multivariate interaction effect (i.e., including all self-report questionnaires of social anxiety traits) in favor of the CBT, i.e. symptom improvement was greater following CBT compared to the ABM control treatment. We also found that state-dependent social anxiety decreased more after CBT. A significant interaction effect (Time × Treatment) was found when covariance was added to the model (MANCOVA; Wilks’s λ=0.678, *F*_2,21_=4.98, *P*=0.017, see also Figure S4); meaning that we included subjective units of discomfort at pre-treatment (see Table S1 and Figure S4).

After receiving CBT, 5 participants (42%, 5/12) did not fulfill the criteria for an SAD diagnosis, but this was not significantly different from the control treatment (ABM: 25%, 3/12; χ^2^=0.72, *P*=0.395).

Treatment effects on structural volume and functional responsivity

Treatment effects (Time × Treatment interactions) across the whole brain are reported in Table S1.

Relation between gray matter volume, functional responsivity and social anxiety

Whole-brain analysis of pre-treatment positive correlations between structural GM volume, or BOLD responsivity, and social anxious symptoms are reported in Table S2. Additionally, positive correlations between decreased GM volume and improved social anxiety are reported in Table S3.

BOLD responsivity did not correlate with social anxiety symptoms at pre-treatment, or when analyzed as change scores following CBT.

Comparisons of structural volume and functional response between SAD participants and healthy controls

Comparisons between SAD participants and healthy controls are shown in Table S4.

Mediation analysis

An additional mediation path analysis using CGI-I as the outcome suggested that decreased GM volume mediated the relationship between declined amygdala BOLD responsivity and symptom improvement, but only at a lenient statistical threshold (*a* × *b* path, indirect effect: *β*=–0.61, CI 95%=–1.29 to 0.06, *P*=0.074, and direct effect, *c’*-path: *β*=0.38, CI 95%=–0.41 to 1.17, *P*=0.347).

Within the ABM control treatment, altered GM volume did not mediate the association between changed BOLD responsivity and decreased social anxiety (or the other way around, i.e., BOLD as the mediator).

**Table S1.** Treatment outcome (before and after treatment) on subjective units of discomfort, demonstrated as estimated means and standard errors.

| **Measure/Time** | | **CBT (*n*=13)** | | | **ABM (*n*=13)** | |  |
| --- | --- | --- | --- | --- | --- | --- | --- |
|  | **Mean** | | **(SE)** | **Mean** | | **(SE)** |  |
| *Subjective unit of discomfort (0-100, min-max)* | | | | | | |  |
| *Baseline anxiety (after acquiring the anatomical image)* | | | | | | |  |
| Pre-treatment | 22.02 | | (0) | 22.02 | | (0) |  |
| Post-treatment | 15.73 | | (4.1) | 10.12 | | (4.1) |  |
|  |  | |  |  | |  |  |
| *Anticipatory speech anxiety (prior to the public speaking task)* | | | | | | |  |
| Pre-treatment | 50.21 | | (0) | 50.21 | | (0) |  |
| Post-treatment | 30.36 | | (5.5) | 45.99 | | (5.5) |  |
|  |  | |  |  | |  |  |

Abbreviations: ABM, attention bias modification; CBT, cognitive behavior therapy

| **Table S2.** Whole-brain treatment effects on structural GM volume and functional responsivity (i.e., pre-treatment vs post-treatment between CBT and the ABM control treatment). | | | | | | | | | | |
| --- | --- | --- | --- | --- | --- | --- | --- | --- | --- | --- |
| Analyses and brain regions MRI | | MNI coordinates | | | | | Maximum *Z* value | | Voxels | *P^uncorr^* |
|  | | x | | y | z | |  |  |  |  |
| *Time × treatment interactions* | | | | | | | | | | |
| L Frontal Mid, BA 8 GM | –20 | | | 23 | | 49 | | 4.54 | 1001 | <0.001 |
| R Frontal Sup Orbital, BA 11 GM | 21 | | | 44 | | –15 | | 4.09 | 349 | <0.001 |
| L Heschl, BA 13 GM | –42 | | | –16 | | 4 | | 3.38 | 14 | <0.001 |
| L Precentral, BA 4, 6 GM | –36 | | | –21 | | 64 | | 4.36 | 849 | <0.001 |
| R Precuneus, BA 7 GM | 2 | | | –48 | | 43 | | 4.36 | 357 | <0.001 |
| R SMA, BA 6 GM | 11 | | | 9 | | 55 | | 3.92 | 365 | <0.001 |
| R Temporal Inferior, BA 20 GM | 51 | | | –13 | | –36 | | 4.02 | 127 | <0.001 |
| L Temporal Mid, ~BA 20 GM | –59 | | | –46 | | –11 | | 3.36 | 11 | <0.001 |
| L Temporal Pole Sup GM | –21 | | | 11 | | –35 | | 3.52 | 74 | <0.001 |
| R Amygdala BOLD | 29 | | | 1 | | –16 | | 3.28 | 15 | <0.001 |
|  |  | |  | |  | |  | |  |  |
|  | | | | | | | | | | |
| Abbreviations: ABM, attention bias modification, BA, Brodmann area; CBT, cognitive behavior therapy; GM, gray matter volume; SMA, supplemental motor area; Sup, superior; uncorr, uncorrected *P*-value | | | | | | | | | | |

| **Table S3.** Whole-brain analysis showing positive correlations between structural GM volume, and anticipatory social anxiety at pre-treatment. | | | | | | | | | |
| --- | --- | --- | --- | --- | --- | --- | --- | --- | --- |
| Analyses and brain regions MRI | | MNI coordinates | | | | | Maximum *Z* value | Voxels | *P^uncorr^* |
|  | | x | | y | | z |  |  |  |
| *Positive correlations* | | | | | | | | | |
| R Caudate GM | 20 | | | | –22 | 18 | 3.26 | 10 | 0.001 |
| L Cuneus, BA 17 GM | –9 | | | | –91 | 1 | 3.81 | 136 | <0.001 |
| R Declive GM | 56 | | | | –61 | –26 | 3.75 | 16 | <0.001 |
| L Inf Temporal Gyrus, BA 37 GM | –50 | | | | –60 | –14 | 3.58 | 54 | <0.001 |
| L Mid Cingulum, BA 31 GM | –2 | | | | –45 | 42 | 3.29 | 34 | 0.001 |
| R Mid Frontal Gyrus, BA 8 GM | 29 | | | | 18 | 51 | 3.32 | 17 | <0.001 |
| R Mid Occipital Gyrus, BA 18 GM | 26 | | | | –91 | 0 | 4.17 | 121 | <0.001 |
| L Mid Temporal Gyrus, BA 21 GM | –66 | | | | –37 | –5 | 3.48 | 170 | <0.001 |
| R Mid Temporal Gyrus, BA 39 GM | 45 | | | | –78 | 20 | 3.90 | 337 | <0.001 |
| R Middle Frontal Gyrus GM | 42 | | | | 18 | 31 | 3.35 | 11 | <0.001 |
| R Occipital Inf, BA 18 GM | 35 | | | | –85 | –5 | 3.25 | 11 | 0.001 |
| R Precuneus, BA 7 GM | 15 | | | | –76 | 47 | 3.82 | 209 | <0.001 |
| L Precuneus, BA 7 GM | –15 | | | | –72 | 51 | 3.81 | 53 | <0.001 |
| R Rectus, BA 13, 47 GM | 17 | | | | 17 | –12 | 3.31 | 14 | <0.001 |
| L Rolandic Oper, BA 13 GM | –48 | | | | –10 | 18 | 3.70 | 136 | <0.001 |
| L Sub–Gyral GM | –41 | | | | –67 | –12 | 3.66 | 102 | <0.001 |
| L Sup Temporal Gyrus, BA 39 GM | –59 | | | | –66 | 28 | 3.52 | 10 | <0.001 |
|  |  | |  | | |  |  |  |  |
|  | | | | | | | | | |
| Abbreviations: BA, Brodmann area; GM, gray matter volume; Inf, inferior; Mid, middle; MNI, Montreal Neurological Institute template; Oper, operculum; uncorr, uncorrected *P*-value | | | | | | | | | |

| **Table S4.** Whole-brain analysis on positive change-change correlations (i.e., decreased GM volume and improved symptoms of anticipatory speech anxiety) | | | | | | | | | | |
| --- | --- | --- | --- | --- | --- | --- | --- | --- | --- | --- |
| Analyses and brain regions MRI | | MNI coordinates | | | | | Maximum *Z* value | | Voxels | *P^uncorr^* |
|  | | x | | y | z | |  |  |  |  |
| *Positive associations* | | | | | | | | | | |
| L Fusiform, BA 18 GM | –26 | | | –73 | | –14 | | 4.06 | 37 | <0.001 |
| L Insula, BA 13 GM | –41 | | | 14 | | 1 | | 4.02 | 62 | <0.001 |
| R Olfactory, BA 47 GM | 27 | | | 11 | | –20 | | 4.14 | 48 | <0.001 |
| R Temporal Superior, BA 22 GM | 60 | | | –4 | | 3 | | 3.97 | 30 | <0.001 |
|  |  | |  | | |  | |  |  |  |
|  | | | | | | | | | | |
| Abbreviations: BA, Brodmann area; CBT, cognitive behavior therapy; GM, gray matter volume; uncorr, uncorrected *P*-value | | | | | | | | | | |

| **Table S5.** Structural and functional response differences between SAD participants (at pre-treatment), and healthy controls | | | | | | | | | | |
| --- | --- | --- | --- | --- | --- | --- | --- | --- | --- | --- |
| Analyses and brain regions MRI | | MNI coordinates | | | | | Maximum *Z* value | | Voxels | *P^uncorr^* |
|  | | x | | y | z | |  |  |  |  |
| *SAD participants > Healthy controls* | | | | | | | | | | |
| R Frontal Inf Oper, ~BA 9 GM | 38 | | | 12 | | 25 | | 4.33 | 107 | <0.001 |
| L Frontal Inf Oper, ~BA 9 GM | -39 | | | 6 | | 22 | | 3.96 | 323 | <0.001 |
| L Frontal Inf Orbital GM | -51 | | | 26 | | -5 | | 3.23 | 11 | 0.001 |
| L Frontal Mid GM | -26 | | | 27 | | 33 | | 3.26 | 11 | 0.001 |
| L Frontal Superior, ~BA 6 GM | -15 | | | -1 | | 72 | | 3.54 | 31 | <0.001 |
| R Frontal Superior, BA 6 GM | 17 | | | -10 | | 72 | | 3.64 | 73 | <0.001 |
| R Frontal Superior, BA 6 GM | 33 | | | -7 | | 63 | | 3.52 | 119 | <0.001 |
| R Frontal Superior, BA 8 GM | 21 | | | 35 | | 43 | | 3.84 | 207 | <0.001 |
| L SMA, ~BA 6 GM | -14 | | | -10 | | 66 | | 3.31 | 21 | <0.001 |
| L Temporal Inf, BA 37 GM | -56 | | | -45 | | -20 | | 3.22 | 11 | 0.001 |
| R Temporal Mid GM | 56 | | | -42 | | -9 | | 3.35 | 23 | <0.001 |
| L Calcarine, ~BA 31 BOLD | –23 | | | –60 | | 9 | | 3.30 | 33 | <0.001 |
| R Calcarine, BA 31 BOLD | 24 | | | –64 | | 15 | | 3.65 | 193 | <0.001 |
| R Cerebellum BOLD | 15 | | | –76 | | –29 | | 3.32 | 31 | <0.001 |
| L Frontal Inf Orb, BA 11, 47 BOLD | –33 | | | 34 | | –13 | | 3.91 | 44 | <0.001 |
| R Putamen BOLD | 29 | | | –15 | | –5 | | 3.90 | 224 | <0.001 |
| L Temporal Mid BOLD | –50 | | | –49 | | 16 | | 3.43 | 39 | <0.001 |
|  |  | | |  | |  | |  |  |  |
| *Healthy controls > SAD participants* | | | | | | | | | | |
| L Calcarine, BA 17 GM | -11 | | | -90 | | 1 | | 4.59 | 527 | <0.001 |
| R Calcarine, BA 17 GM | 14 | | | -87 | | 3 | | 3.20 | 12 | 0.001 |
|  |  | |  | |  | |  | |  |  |
|  | | | | | | | | | | |
| Abbreviations: BA, Brodmann area; GM, gray matter volume; Inf, inferior; Mid, middle; MNI, Montreal Neurological Institute template; Orb, orbital; SAD, social anxiety disorder; SMA, supplemental motor area; uncorr, uncorrected *P*-value | | | | | | | | | | |

**
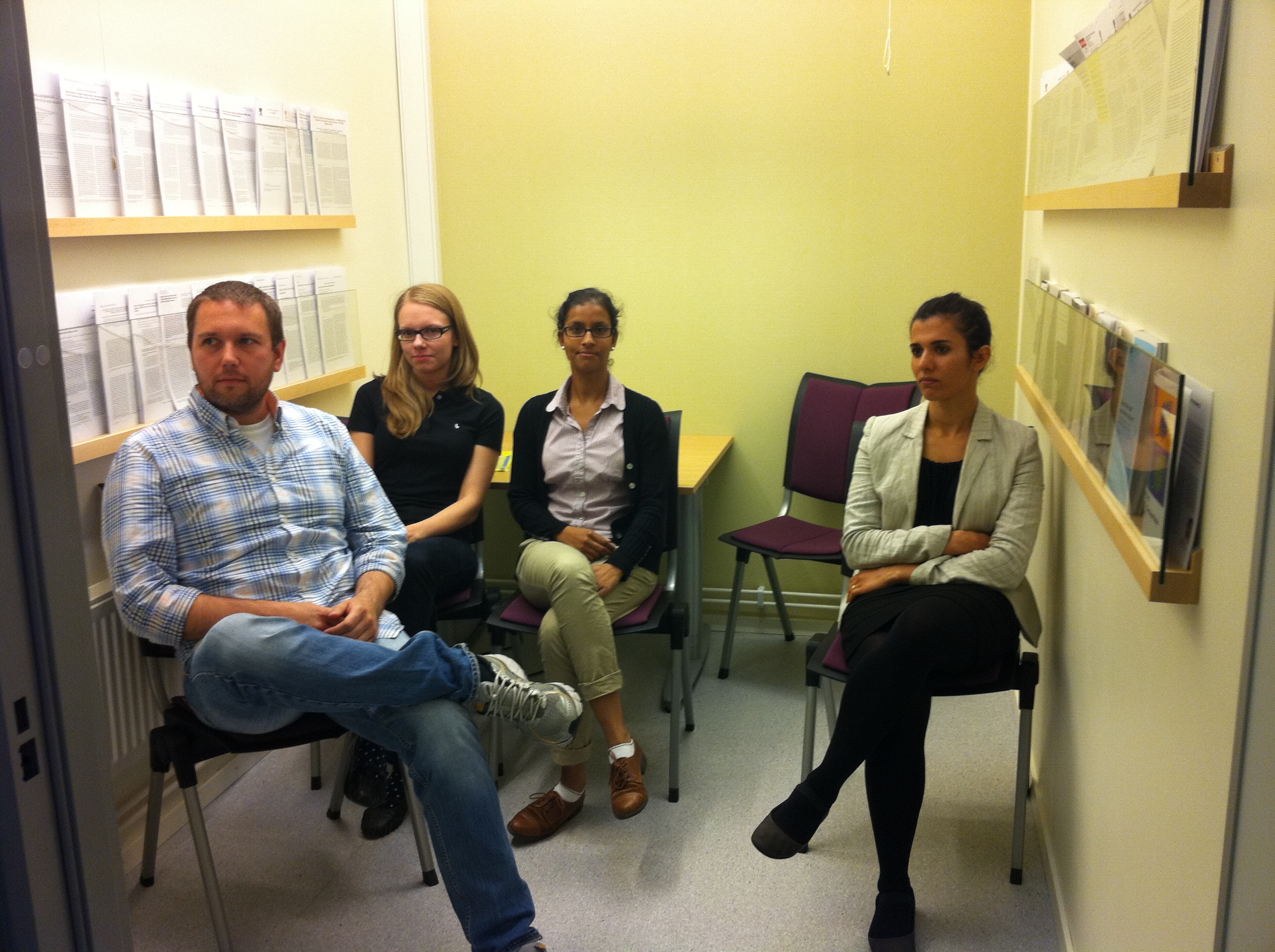

Figure S1.** Photograph of the room where the public speaking task took place. The experimenter of the study (1^st^ author) was always seated in the back (i.e., the empty chair). Anticipatory speech anxiety was rated before the participants entered the room.

**
Figure S2.** Flow-chart demonstrating randomization and dropouts in the clinical imaging trial

Abbreviations: CGI-I, Clinically Global Impression-Improvement scale; MADRS-S, Montgomery Åsberg Depression Rating Scale – Self-rating version; MRI, magnetic resonance imaging; SAD, social anxiety disorder; SCID, structured clinical interview for diagnostic and statistical manual of mental disorders, 4^th^ version; SPSQ, Social Phobia Screening Questionnaire

**Figure S3.** In the BOLD-fMRI experimental task the participants were instructed to read sentences and press a button as a confirmation. The sentences contained criticism targeting the self or others. Fixation crosses were randomly interspersed between the sentences, and each sentence and fixation cross was separated by a cross or circle

**
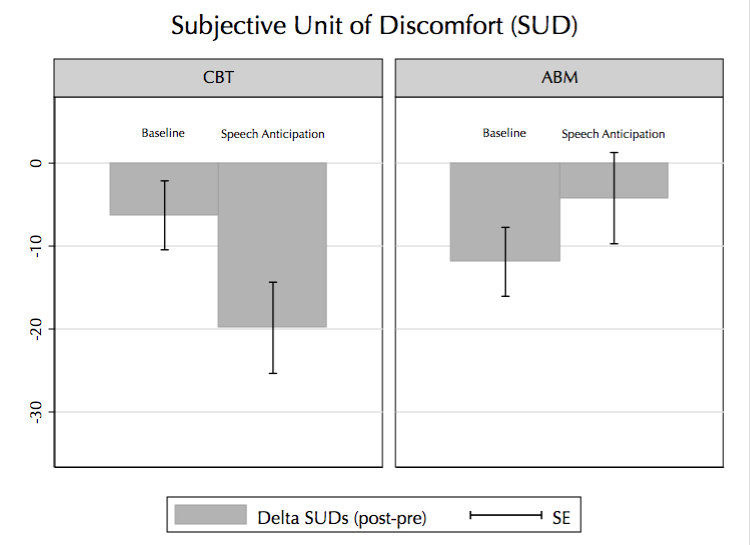
**

**Figure S4.** Figure showing that symptom improvement (pre-treatment > post-treatment) on state-dependent social anxiety was in favor of cognitive behavior therapy (CBT), relative to the attention bias modification (ABM). “Baseline” refers to anxious ratings after the acquisition of the anatomical image, whereas “Speech anticipation” refers to anxiety ratings obtained before the public speaking task. Error bars represent standard errors.

SUPPLEMENTARY References

1. Zaider TI, Heimberg RG, Fresco DM, Schneier FR, Liebowitz MR. Evaluation of the Clinical Global Impression Scale among individuals with social anxiety disorder. *Psychol Med* 2003; **33**(4)**:** 611-622.

2. First M, Gibbon M, Spitzer R, Williams J. *Structured clinical interview for DSM–IV axis I disorders (SCID–I)*. American Psychiatric Press: Washington, 1997.

3. Wolpe J. *The practice of behavior therapy*. 4th edn. Pergamon Press: New York, 1990.

4. Månsson KN, Frick A, Boraxbekk CJ, Marquand AF, Williams SC, Carlbring P *et al.* Predicting long-term outcome of Internet-delivered cognitive behavior therapy for social anxiety disorder using fMRI and support vector machine learning. *Transl Psychiatry* 2015; **5:** e530.

5. Blair K, Geraci M, Devido J, McCaffrey D, Chen G, Vythilingam M *et al.* Neural response to self- and other referential praise and criticism in generalized social phobia. *Arch Gen Psychiatry* 2008; **65**(10)**:** 1176-1184.

6. Andersson G, Carlbring P, Furmark T, Group SOFIER. Therapist experience and knowledge acquisition in internet-delivered CBT for social anxiety disorder: a randomized controlled trial. *PLoS One* 2012; **7**(5)**:** e37411.

7. Andersson G, Carlbring P, Holmström A, Sparthan E, Furmark T, Nilsson-Ihrfelt E *et al.* Internet-based self-help with therapist feedback and in vivo group exposure for social phobia: a randomized controlled trial. *J Consult Clin Psychol* 2006; **74**(4)**:** 677-686.

8. Carlbring P, Gunnarsdottir M, Hedensjo L, Andersson G, Ekselius L, Furmark T. Treatment of social phobia: randomised trial of internet-delivered cognitive-behavioural therapy with telephone support. *Br J Psychiatry* 2007; **190:** 123-128.

9. Furmark T, Carlbring P, Hedman E, Sonnenstein A, Clevberger P, Bohman B *et al.* Guided and unguided self-help for social anxiety disorder: randomised controlled trial. *Br J Psychiatry* 2009; **195**(5)**:** 440-447.

10. Clark DM, McManus F. Information processing in social phobia. *Biol Psychiatry* 2002; **51**(1)**:** 92-100.

11. Clark DM, Wells A. A cognitive model of social phobia. In: R. Heimberg ML, D. A. Hope, & F. R. Schneier (ed). *Social phobia: Diagnosis, assessment and treatment*. Guilford Press: New York, 1995, pp 69-93.

12. Furmark T, Holmström A, Sparthan E, Carlbring P, Andersson G. *Social fobi - Effektiv hjälp med kognitiv beteendeterapi [Social Anxiety - Effective treatment using cognitive behavioural therapy]*. 2nd edn. Liber: Stockholm, 2006.

13. Carlbring P, Apelstrand M, Sehlin H, Amir N, Rousseau A, Hofmann SG *et al.* Internet-delivered attention bias modification training in individuals with social anxiety disorder--a double blind randomized controlled trial. *BMC Psychiatry* 2012; **12**(1)**:** 66.

14. MacLeod C, Mathews A. Cognitive bias modification approaches to anxiety. *Annu Rev Clin Psychol* 2012; **8**(1)**:** 189-217.

15. Månsson KNT, Carlbring P, Frick A, Engman J, Olsson C-J, Bodlund O *et al.* Altered neural correlates of affective processing after internet-delivered cognitive behavior therapy for social anxiety disorder. *Psychiatry Res* 2013; **214**(3)**:** 229-237.
